# Common genetic variation and novel loci associated with volumetric mammographic density

**DOI:** 10.1186/s13058-018-0954-6

**Published:** 2018-04-17

**Authors:** Judith S. Brand, Keith Humphreys, Jingmei Li, Robert Karlsson, Per Hall, Kamila Czene

**Affiliations:** 1 Department of Medical Epidemiology and Biostatistics, Karolinska Institutet, Nobels Väg 12A, 171 77 Stockholm, Sweden; 2 Human Genetics, Genome Institute of Singapore, Singapore, Singapore

**Keywords:** Mammographic density, Genetic association study, Heritability

## Abstract

**Background:**

Mammographic density (MD) is a strong and heritable intermediate phenotype of breast cancer, but much of its genetic variation remains unexplained.

**Methods:**

We conducted a genetic association study of volumetric MD in a Swedish mammography screening cohort (*n* = 9498) to identify novel MD loci. Associations with volumetric MD phenotypes (percent dense volume, absolute dense volume, and absolute nondense volume) were estimated using linear regression adjusting for age, body mass index, menopausal status, and six principal components. We also estimated the proportion of MD variance explained by additive contributions from single-nucleotide polymorphisms (SNP-based heritability [*h*^2^_SNP_]) in 4948 participants of the cohort.

**Results:**

In total, three novel MD loci were identified (at *P* < 5 × 10^− 8^): one for percent dense volume (*HABP2*) and two for the absolute dense volume *(INHBB, LINC01483)*. *INHBB* is an established locus for ER-negative breast cancer, and *HABP2* and *LINC01483* represent putative new breast cancer susceptibility loci, because both loci were associated with breast cancer in available meta-analysis data including 122,977 breast cancer cases and 105,974 control subjects (*P* < 0.05). *h*^2^_SNP_ (SE) estimates for percent dense, absolute dense, and nondense volume were 0.29 (0.07), 0.31 (0.07), and 0.25 (0.07), respectively. Corresponding ratios of *h*^2^_SNP_ to previously observed narrow-sense *h*^2^ estimates in the same cohort were 0.46, 0.72, and 0.41, respectively.

**Conclusions:**

These findings provide new insights into the genetic basis of MD and biological mechanisms linking MD to breast cancer risk. Apart from identifying three novel loci, we demonstrate that at least 25% of the MD variance is explained by common genetic variation with *h*^2^_SNP_/*h*^2^ ratios varying between dense and nondense MD components.

**Electronic supplementary material:**

The online version of this article (10.1186/s13058-018-0954-6) contains supplementary material, which is available to authorized users.

## Background

Mammographic density (MD) refers to the amount of fibroglandular dense and fatty nondense tissues in the breast, which appear white and black, respectively, on an X-ray mammogram. Women with high MD (i.e., a high amount of fibroglandular dense tissue) are at four to sixfold increased risk of developing breast cancer compared with women having nondense or fatty breasts [1]. Despite being an important determinant of breast cancer risk, the biological mechanisms determining tumor development in women with highly dense breasts are not well understood. Understanding the genetic basis of MD and how its associated loci are related to breast cancer risk may shed light on the processes that are responsible for the transformation of mammographic dense tissue into tumor tissue. Moreover, as a strong intermediate phenotype for breast cancer [2], genetic association studies of MD have the potential to identify new breast cancer susceptibility loci.

Family-based studies have estimated that approximately 60–70% of the variance in MD is explained by additive genetic effects, which is considerably higher than the narrow-sense heritability estimate reported for breast cancer (*h*^2^ = 30–40%). To date, genome-wide association studies (GWAS) [3–7] have identified MD-associated single-nucleotide polymorphisms (SNPs) at 13 loci (1q12.21, *AREG*, *PRDM6, TAB2*, *CCDC170/ESR1, ESR1*, 8p11.23, *ZNF365*, *LSP1, IGF1*, 12q24, *TMEM184B, and SGSM3/MKL1*) across the genome, most of which have also been associated with breast cancer. However, additive effects of these genome-wide significant SNPs explain only a small fraction of the total MD variance (< 5%). This discrepancy in explained variance or “missing heritability” has been attributed to various factors, including the presence of large numbers of common variants with small effects, rare variants with large effects not tagged by common SNPs on genotyping arrays and possible inflation of narrow-sense *h*^2^ estimates if family resemblance is influenced by nonadditive effects, shared environmental effects, and/or gene-environment interactions. Although the exact contribution of each of these possible explanations is difficult to determine, the contribution of common genetic variation to MD variance (SNP-based heritability or [*h*^2^_SNP_]) can be assessed rather easily in genome-wide association data and can provide insights into the heritability fraction that remains to be identified in future larger-scale GWAS.

In the present study, we conducted a large-scale genetic association analysis of volumetric MD to identify novel MD loci. We further sought to unravel the genetic basis of MD through estimating the proportion of MD variance attributable to common SNPs. Previous GWAS of MD have relied mainly on area-based or 2D MD measures derived from film mammograms using labor-intensive semiautomated methods [8]. For this analysis, we used volumetric MD measures derived from digital mammograms using a fully automated method [9].

## Methods

### Study design and participants

A genetic association meta-analysis was conducted within the Karolinska Mammography Project for Risk Prediction of. Breast Cancer (Karma), a screening-based cohort of women attending one of four mammography units in the Swedish national screening program between 2011 and 2013 [10]. Participants responded to a web-based questionnaire, donated blood, and gave permission for storage of raw full-field digital mammograms. Genotyping data passing quality control metrics were available for two subcohorts of Karma participants with MD measurements, including 5827 women genotyped with the OncoArray and 4021 women genotyped with the Infinium iSelect genotyping array (Illumina, San Diego, CA, USA) of the Collaborative Oncological Gene-environment Study (iCOGS) (*see details below*). All women were free of cancer at the time of blood sampling and had no history of breast enlargement, reduction, or surgery.

### Volumetric MD assessment

MD in Karma was estimated from the mediolateral oblique view of baseline screening mammograms using Volpara™ version 1.5.0 (Mātakina, Wellington, New Zealand), which is a fully automated method for volumetric MD estimation approved by the U.S. Food and Drug Administration. Volpara MD measures show good agreement with breast magnetic resonance imaging data [9] and have been validated as being predictive of breast cancer risk [11, 12]. Technical details of the Volpara software have been described in detail elsewhere [9]. In brief, the algorithm computes the thickness of dense tissue at each pixel using the X-ray attenuation of an entirely fatty region as an internal reference. The absolute dense volume (cm^3^) is measured by integrating the dense thickness at each pixel over the whole mammogram, and the total breast volume (cm^3^) is derived by multiplying the breast area by the recorded breast thickness, with an appropriate correction for the breast edge. From these measures, the absolute nondense volume (cm^3^) and the percentage of the breast covered by dense tissue (%) can be obtained. The mean volumetric measurement of the left and right breast was taken for all analyses. Distributions of the three MD measures (percent dense volume, absolute dense volume, and absolute nondense volume) in the Karma-OncoArray and Karma-iCOGS genotyping cohorts are presented in Additional file 1: Figure S1.

### Covariates

The following covariates were extracted from the Karma web-based questionnaire administered at baseline: age at study entry, menstrual and reproductive factors, and body mass index (BMI) as estimated from self-reported height and weight. Menopausal status was defined according to reported menstruation status, previous oophorectomy, and age. Women were considered postmenopausal if they had not menstruated during the past year, had a history of oophorectomy, or were above the age of 55 years.

### Genotyping, quality control, and imputation

Whole-blood samples were genotyped using the OncoArray (*see* https://support.illumina.com/downloads/infinium-oncoarray-500k-v1-0-product-files.html for detailed information on array design), which covers more than 500,000 variants, or the iCOGS array (http://ccge.medschl.cam.ac.uk/research/consortia/icogs/), with over 200,000 variants. Both arrays use custom bead chips with markers of interest for breast and other cancers, fine-mapping of known susceptibility loci, and markers associated with quantitative phenotypes that correlate with common cancers. The OncoArray is the successor of iCOGS and therefore denser, including additional variants of sequencing studies and a more informative backbone of approximately 260,000 SNPs that provides genome-wide coverage of most common variants. More details on both genotyping arrays can be found elsewhere [13, 14].

Samples were excluded from analysis for any of the following reasons: gender discordance according to array data, chromosomal anomalies (XXY or XO), extremely low or high heterozygosity (4.89 SD from the mean for the ethnicity), discordant duplicate pairs, first-degree relatives, and low call rates **(***see* Additional file 1: Table S1 for number of individuals excluded per criterion**)**. Standard SNP quality control was performed in PLINK version 1.9 [15], and SNPs with minor allele frequency (MAF) < 0.01 or deviation from Hardy-Weinberg equilibrium at *P* < 1 × 10^− 6^ were excluded. To increase resolution and coverage, nongenotyped SNPs were imputed using the 1000 Genomes Project March 2012 release as the reference [16]. Data were imputed in a two-stage procedure using SHAPEIT to derive phased genotypes and IMPUTE version 2 to perform the imputation on the phased data [17]. Postimputation quality control was based on the IMPUTE info score, and SNPs with a score < 0.80 or MAF < 0.01 were excluded, resulting in a total of 8.5 million SNPs for analyses.

### SNP association analyses

All three mammographic phenotypes (percent dense volume, absolute dense volume, and absolute nondense volume) were log-transformed to approximate the normal distribution (Additional file 1: Figure S2). SNP association analyses were performed in each genotyping cohort using linear regression and assuming an additive genetic model. Imputed SNPs were analyzed using a score test that employs allele dosages instead of hard genotype calls [18]. Population stratification was assessed using principal component analysis (PCA) in PLINK version 1.9 [15], and analyses were adjusted for age (years), BMI (kg/m^2^), menopausal status (postmenopausal vs. premenopausal), and six cohort-specific PCA scores to account for population substructure. β-Coefficients of the two genotyping cohorts were meta-analyzed using a fixed effects model as implemented in METAL [19] with the Cochran’s *Q* statistic used as a test for between-study heterogeneity. Regional association plots of identified variants were generated using LocusZoom [20] with the 400-kb region centered on the lead SNP.

### Functional annotation and breast cancer association analyses of newly identified variants

We used several web tools for the functional annotation of the lead SNPs and their proxies (*r*^2^ > 0.80). We checked for potential regulatory functions using the HaploReg [21] and Regulome [22] databases, based on Encyclopedia of DNA Elements data [23] for the human mammary epithelial cell (HMEC) and mammary tumor cell (MCF-7) lines. We further searched the publicly available Genotype-Tissue Expression Project database (http://www.gtexportal.org/) for evidence of cis expression quantitative trait loci (eQTL) at each locus in mammary tissue samples (*n* = 183).

Associations between newly identified MD variants and breast cancer risk (overall and by estrogen receptor [ER] status) were checked using data from the Breast Cancer Association Consortium (BCAC), including 122,977 breast cancer cases and 105,974 control subjects (http://bcac.ccge.medschl.cam.ac.uk/bcacdata/oncoarray/gwas-icogs-and-oncoarray-summary-results/) [14]. Information on ER status was available for 90,969 cases, of which 69,501 were ER-positive and 21,468 were ER-negative. We also verified associations with MD loci identified by previous GWAS [3–7], with the threshold of statistical significance set to *P* = 0.05/13 (number of loci) = 3.85 × 10^− 3^.

#### SNP-based heritability analyses

The heritability of a trait is defined as the proportion of phenotypic variance explained by genetic factors, including additive genetic effects, dominant effects, and epistasis. Narrow-sense heritability *(h*^*2*^) refers to the variance component corresponding to additive genetic effects and can be estimated by exploring phenotypic similarities between relatives in family or twin studies.

Genome-wide complex trait analysis (GCTA) software [24, 25] was used to estimate the proportion of variance explained by additive effects of all SNPs. The interpretation of *h*^2^_SNP_ estimated with GCTA is different from the *h*^2^ obtained from traditional family-based studies, because the latter captures variance due to additive effects of all causal variants in the genome (including rare variants) and can be inflated if family resemblance is influenced by nonadditive genetic effects (dominance and epistasis or gene-gene interactions), shared environmental effects, and/or gene-environment interactions. Our GCTA analysis was conducted in the Karma-OncoArray cohort only, because the iCOGS array has insufficient coverage to obtain reliable genome-wide estimations of SNP-based heritability. First, pairwise genetic relationships between all individuals were calculated, followed by estimation of the additive genetic variance explained by all SNPs using restricted maximum likelihood analysis. We excluded one individual per pair whose estimated coefficient of relatedness was > 0.025 (which corresponds to third- or fourth-degree cousins), in order to prevent confounding by possible shared environmental effects and effects of causal variants that are not tagged by the SNPs. This resulted in a study population of 4948 women for which *h*^2^_SNP_ could be estimated for percent, absolute dense, and nondense MD. The covariates included were the same as those for the individual SNP analyses, and estimates of SNP-based heritabilities were compared with corresponding *h*^2^ estimates previously reported in a study of siblings in Karma [26].

## Results

Table 1 summarizes the characteristics of the Karma-OncoArray and Karma-iCOGS genotyping cohorts. Karma-OncoArray women were older and more often postmenopausal than women of the Karma-iCOGS cohort. Because of the older age distribution, mean MD levels were lower in the Karma-OncoArray cohort (*see also* Additional file 1: Figure S1 for the MD distributions per genotyping cohort). No major differences in BMI or family history of breast cancer were observed between the two cohorts.

**Table 1:** Characteristics of the Karma study population

	Karma-OncoArray (*n* = 5827)	Karma-iCOGS (*n* = 4021)
Age, years, mean (SD)	60.2 (9.2)	53.6 (9.4)
Body mass index, kg/m^2^, mean (SD)	25.5 (4.2)	25.3 (4.2)
Menopausal status, % (*n*)		
Premenopausal	22.8 (1327)	49.0 (1969)
Postmenopausal	77.2 (4500)	51.0 (2052)
Percent dense volume (%), median (IQR)	6.7 (5.2)	8.4 (6.5)
Dense volume, cm^3^, median (IQR)	53.4 (31.1)	60.3 (36.9)
Nondense volume, cm^3^, median (IQR)	744 (616)	676 (581)
Family history of breast cancer, % (*n*)	13.7 (769)	12.0 (482)

*Karma* Karolinska Mammography Project for Risk Prediction of. Breast Cancer

Descriptive statistics of the Karma-OncoArray and Karma-iCOGS genotyping cohorts

*Abbreviations*: *SD* standard deviation, *IQR* interquartile range

Quantile-quantile plots of the genome-wide meta-analysis results for each MD measure are presented in Additional file 1: Figure S3. Overall, genomic inflation factors showed little or no evidence for inflation (λ for percent dense volume, absolute dense volume, and absolute nondense volume were 1.02, 1.03, and 1.02, respectively). Additional file 1: Figure S4 shows the Manhattan plots displaying the log_10_-transformed *P* values for each SNP. In total, we identified eight independent loci for any MD measure (*ZNF365*, *TAB2,* *HABP2*, *INHBB, AREG, LINC01483*, *MKL1,* and 8p11.23) at *P* < 5 × 10^− 8^ (Additional file 1: Table S2), three of which were novel (*HABP2, INHBB, and LINC01483*) and the remaining five (*ZNF365, TAB2, AREG, 8p11.23, and MKL1*) of which were previously reported to be associated with MD [3, 5, 7, 27] in the same directions as observed in the present analysis. We further replicated five of eight MD loci identified by previous GWAS (1q12.21*, ESR1, CCDC170/ESR1, IGF1, and SGSM3/MKL1*) beyond the loci reaching genome-wide significance in pooled analysis (Additional file 1: Table S3). The association between *PRDM6* and percent dense volume was marginally significant (*P* < 0.05), with direction of effect being consistent with that reported for percent dense area [5]. No significant associations of *TMEM184B* and *LSP1* with volumetric MD were observed. Altogether, the newly identified and established MD loci explained only small fractions of the total variance in percent dense (1.6%), absolute dense (2.8%), and absolute nondense (0.5%) volume.

**Fig. 1:**
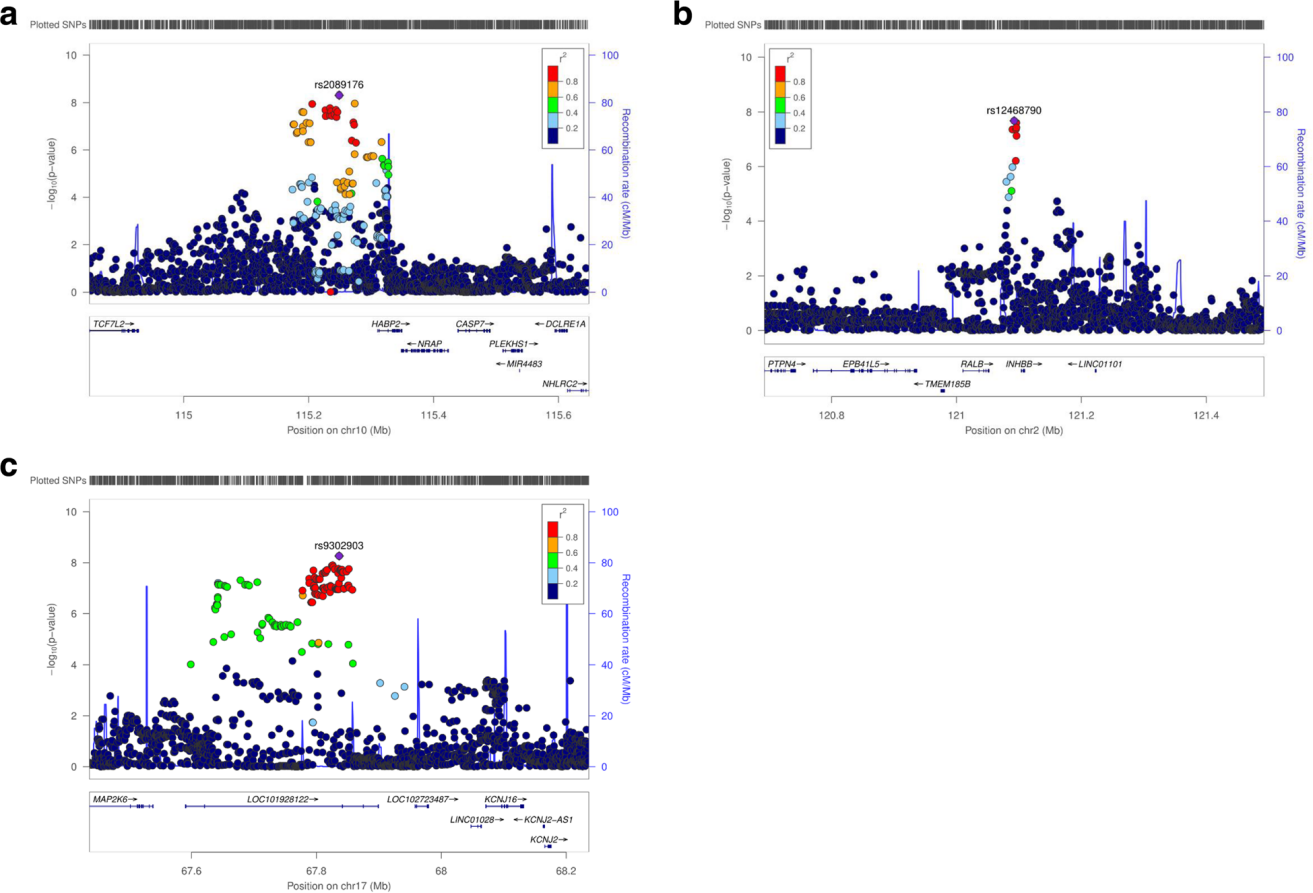
Regional association plots for newly identified mammographic density loci. Regional plots of single-nucleotide polymorphisms (SNPs) associated with volumetric mammographic density measures. Lead SNPs are shown in *purple*: *A* = percent dense volume (rs2089176); *B* = absolute dense volume (rs12468790); *C* = absolute dense volume (rs9302903). *Circles* denote imputed SNPs; *squares* denote genotyped SNPs. Colors indicate the extent of linkage disequilibrium. Genetic recombination rates are estimated using 1000 Genomes EUR sample and are presented as the *light blue line*. Physical positions are based on NCBI Genome Reference Consortium Human Build 37 (GRCh37). Plots were generated using LocusZoom [20]. *LOC101928122* stands for *LINC01483*

The lead SNPs at the three newly identified MD loci *(HABP2, INHBB, and LINC01483)* are summarized in Table 2 with corresponding regional association plots in Fig. 1. SNP rs2089176 (10q25.3) associated with percent dense volume lies 120 kb upstream of its closest gene, *HABP2*. The two index SNPs associated with absolute dense volume span noncoding parts of the genome. SNP rs12468790 at 2q14.2 is located 11 kb upstream of the *INHBB* gene **(**Fig. 1**)** and rs9302903 at chromosome 17q24.3 falls within a long intergenic noncoding RNA gene (long intergenic non-protein coding RNA 1483 [*LINC01483]*) in a region flanking the mitogen-activated protein kinase 6 *(MAP2K6)* and the potassium voltage-gated channel subfamily J, member 16 *(KCNJ16)* genes. Of note, a variant (rs4849887) downstream of the *INHBB* gene but not in linkage disequilibrium (LD) with rs12468790 is an established breast cancer susceptibility variant [28, 29]. The association of rs12468790 with absolute dense volume remained significant in conditional analysis adjusting for rs4849887 (*P* = 1.5 × 10^− 8^), supporting the presence of two independent signals at this locus.

**Table 2:** Novel loci associated with volumetric mammographic density

						Karma-OncoArray (*n* = 5827)			Karma-iCOGS (*n* = 4021)			Karma pooled MD analysis (*N* = 9848)		
Locus	Base pair	Lead SNP	Gene	A1	A2	MAF	β (SE)	*P value*	MAF	β (SE)	*P value*	β (SE)	*P* overall	*P value* for heterogeneity
Percent dense volume														
10q25.3	115,248,851	rs2089176	*HABP2*	A	G	0.41	− 0.05 (0.01)	3.2 × 10^− 4^	0.41	− 0.08 (0.02)	1.4 × 10^− 6^	− 0.07 (0.01)	4.1 × 10^− 9^	0.20
Absolute dense volume														
2q14.2	121,092,388	rs12468790	*INHBB*	A	G	0.39	0.08 (0.02)	2.4 × 10^− 5^	0.36	0.08 (0.02)	2.3 × 10^− 4^	0.08 (0.01)	2.1 × 10^− 8^	0.84
17q24.3	67,836,371	rs9302903	*LINC01483*	T	C	0.08	− 0.12 (0.03)	7.5 × 10^− 5^	0.08	− 0.17 (0.04)	1.3 × 10^− 5^	− 0.14 (0.02)	5.9 × 10^− 9^	0.36

*Abbreviations: iCOGS* Illumina iSelect genotyping array of the Collaborative Oncological Gene-environment Study, *Karma* Karolinska Mammography Project for Risk Prediction of. Breast Cancer, *SNP* Single-nucleotide polymorphism, *A1* Major allele, *A2* Minor allele, *MAF* Minor allele frequency

Gene refers to nearest gene. All lead SNPs were imputed: INFO scores Karma-OncoArray: rs2089176 (0.99), rs12468790 (0.96), rs9302903 (0.99)

For all newly identified loci, no predicted functional eQTL consequences were found in breast mammary tissue, but several variants in strong LD with rs2089176 *(HABP2)* mapped to a region defined by DNase and enhancer histone marks in HMECs and MCF-7 cells. In particular, one variant (rs1472751) was identified in a region binding CCCTC-binding factor (CTCF) in HMECs and MCF-7 cells, as well as GATA3 in MCF-7 cells. CTCF is a transcription factor that regulates a wide range of genes involved in growth, proliferation, differentiation, and apoptosis; GATA3 is the most abundant transcription factor in luminal epithelial cells and plays a key role in normal development of the mammary gland. Two variants in LD with rs9302903 *(LINC01483)* also mapped to DNase and enhancer histone marks in HMECs. No evidence of regulatory function was found for rs12468790 (*INHBB*) (Additional file 1: Table S4).

Associations of the three MD loci with breast cancer risk are summarized in Table 3. rs2089176 (*HABP2*) and rs9302903 *(LINC01483*) were associated with breast cancer risk in BCAC meta-analysis data (rs2089176 OR [95% CI] per minor allele increase = 0.98 [0.97–0.99], *P* = 0.01; rs9302903 OR [95% CI] per minor allele increase = 0.96 [0.94–0.99], *P* = 1.2 × 10^− 3^) in a direction consistent with that observed in the pooled MD analysis for percent dense volume [rs2089176 β [SE] per minor allele increase = − 0.07 (0.01)] and absolute dense volume [rs9302903: β [SE] per minor allele increase = − 0.14 (0.02)]. rs12468790 *(INHBB)* was also associated with breast cancer but in a direction opposite to its effect on MD. Each minor allele increase at rs12468790 resulted in a larger absolute dense volume (β [SE] = − 0.03 [0.01]) but a lower odds of breast cancer (OR [95% CI] = 0.97 [0.96–0.98], *P* = 4.8 × 10^− 6^). Stratified analyses by breast cancer subtype revealed that the association of rs12468790 *(INHBB)* with breast cancer was stronger for ER-negative (OR [95% CI] per minor allele increase = 0.93 [0.91–0.95], *P* = 1.5 × 10^− 9^) than ER-positive tumors (OR [95% CI] = 0.98 [0.96–0.99], *P* = 3.8 × 10^− 3^).

**Table 3:** Associations of newly identified mammographic density loci with breast cancer risk, overall and stratified by estrogen receptor status

						Karma		BCAC						
						Pooled MD analysis		MAF	Breast cancer overall		Breast cancer ER−		Breast cancer ER+	
Locus	Base pair	Lead SNP	Gene	A1	A2	β (SE)	*P* overall		OR (95% CI)	*P value*	OR (95% CI)	*P value*	OR (95% CI)	*P value*
Percent dense volume														
10q25.3	115,248,851	rs2089176	*HABP2*	A	G	− 0.07 (0.01)	4.1 × 10^− 9^	0.36	0.98 (0.97–0.99)	0.01	1.00 (0.98–1.03)	0.70	0.97 (0.96–0.99)	5.3 × 10^− 4^
Absolute dense volume														
2q14.2	121,092,388	rs12468790	*INHBB*	A	G	0.08 (0.01)	2.1 × 10^− 8^	0.40	0.97 (0.96–0.98)	4.8 × 10^− 6^	0.93 (0.91–0.95)	1.5 × 10^− 9^	0.98 (0.96–0.99)	3.8 × 10^− 3^
17q24.3	67,836,371	rs9302903	*LINC01483*	T	C	− 0.14 (0.02)	5.9 × 10^− 9^	0.09	0.96 (0.94–0.99)	1.1 × 10^− 3^	0.98 (0.94–1.02)	0.26	0.95 (0.93–0.98)	1.5 × 10^− 4^

*Abbreviations*: *BCAC* Breast Cancer Association Consortium, *SNP* Single-nucleotide polymorphism, *A1* Major allele, *A2* Minor allele, *MAF* Minor allele frequency, *ER*− Estrogen receptor-negative, *ER+* Estrogen receptor-positive

MAF in BCAC as observed in genotyped control subjects (Europeans only). Gene refers to nearest gene

Finally, we estimated the MD variance attributable to common genetic variants in the Karma-OncoArray cohort. SNP-based heritability (*h*^2^_SNP_) estimates for percent dense, absolute dense, and absolute nonvolume were 0.29 (SE = 0.07), 0.31 (SE = 0.07), and 0.25 (SE = 0.07) respectively, with evidence of a moderate genetic overlap between the absolute dense and nondense volume (*r*_g_ = 0.45, SE = 0.14). Given the narrow-sense *h*^2^ estimates observed in previous sibling analyses in Karma (*h*^2^ = 0.63 [SE = 0.06], 0.43 [SE = 0.06], and 0.61 [SE = 0.06] for percent, absolute dense, and absolute nondense, volume, respectively [26]), the ratio of *h*^2^_SNP_ to these narrow-sense *h*^2^ estimates was substantially higher for the absolute dense volume (0.72) than for percent dense (0.46) and absolute nondense (0.41) volume.

## Discussion

In this study, we identified three novel MD loci at genome-wide significance (percent dense volume [*HABP2]* and absolute dense volume [*INHBB, LINC01483*]), of which two (*HABP2, LINC01483*) represent putative novel breast cancer susceptibility variants. We further demonstrate that at least 25% of the variance in volumetric MD is explained by common genetic variants, and that the ratio of SNP-based to narrow-sense heritability differs between the absolute dense and nondense volume.

All identified MD loci map to noncoding areas of the genome with nearby genes that have been linked to breast cancer etiology and/or mammary development. SNP rs2089176 upstream of the *HABP2* gene at 10q25.3 spans a region with CTCF and GATA3 transcription factor binding sites. The *HABP2* gene encodes for an extracellular serine protease that, upon binding to its ligand hyaluronic acid (HA), activates degradation of the extracellular matrix, including disruption of the endothelium, promoting tumor angiogenesis and cancer metastasis. In patients with breast cancer, a high HA content in malignant epithelial and stromal cells [30, 31] and elevated circulating HA levels [32, 33] have been associated with poor tumor differentiation, unfavorable prognostic features, and a reduction in MD. The protein encoded by *INHBB,* a subunit of both inhibin and activin (two glycoproteins belonging to the transforming growth factor-β superfamily), is critical in normal mammary development because its loss is accompanied by retarded ductal elongation and alveolar morphogenesis as well as failure of lactation [34]. Variants upstream of *INHBB* have previously been associated with bra cup size in a large European cohort of 16,000 women [35] but not with mammographic dense tissue specifically. Interpretation of the MD locus at 17q24.3 (*LINC01483*) is more speculative, because long intergenic noncoding RNAs are not well-characterized. Functional annotation revealed several correlated variants mapping to enhancer histone marks in HMECs, which may indicate a role of this locus in the expression of nearby genes (*MAP2K6* and *KCNJ16*), of which *MAP2K6* is known to play a role in the malignant transformation of breast epithelial cells [36, 37].

Finding genetic variants associated with both MD and breast cancer increases the understanding of biological pathways shared by both traits. Moreover, discovery of new MD loci in genome-wide approaches may provide information about putative novel susceptibility loci for breast cancer and/or its subtypes. Of the newly identified loci, two *(HABP2* and *LINC01483*) showed associations with MD that are consistent with the MD-breast cancer risk association, suggesting that part of their effect on breast cancer susceptibility is mediated through a directionally consistent change in MD. The association of rs12468790 (*INHBB*) with the absolute dense volume, however, was not consistent with the MD-breast cancer risk association. Although the exact nature of this conflicting direction of effect is unknown, it may represent potential mediation by proxies closely related to MD that exert differential effects on breast cancer risk. *INHBB*, for instance, is highly expressed in adipose tissue [38] and has previously been linked to total breast size, a strong correlate of the absolute nondense volume [35, 39]. Albeit not reaching statistical significance, the *INHBB* variant was weakly associated with the absolute nondense volume in our study (rs12468790: β [SE] per minor allele increase = 0.03 [0.01], *P* = 0.01), and recent data indicate that dense and nondense adipose tissues are associated with breast cancer in opposite directions [40, 41]. The stronger association of this variant with ER-negative than ER-positive breast cancers may also point to alternative mechanisms linking *INHBB* to breast cancer than a causal path acting through mammographic dense tissue.

Apart from identifying novel MD loci, the present study replicates many loci identified by previous GWAS of area-based MD measures, except for *TMEM184B*, *LSP1*, 8p11.23, and 12q24 [4, 5]. Because our study concerns the volume of dense tissue in the breast rather than its projection, some inconsistency with previous GWAS is anticipated because volumetric and area-based measures reflect slightly different aspects of MD [7, 11, 26, 42]. Area-based and volumetric measures show good levels of agreement for percent MD (*r* ≈ 0.9), but less so for absolute MD (*r* ≈ 0.5) [11, 12]. Nevertheless, both percent and absolute MD measures show equivalent associations with breast cancer risk [11, 43, 44], regardless of measurement type. Lack of association in the Karma-iCOGS cohort, which included larger numbers of premenopausal women, may also reflect differential SNP effects over the life course. Previous GWAS were based largely on postmenopausal women, and some of the loci identified by these efforts and in the Karma-OncoArray cohort may not generalize to younger women. Differences in study design may further account for some of the differences observed. In contrast to previous GWAS, our study population did not include breast cancer cases with prediagnostic MD measurements. This reduces the likelihood of spurious associations due to confounding by breast cancer but may also have resulted in limited power to identify MD loci because of less extreme MD variation in cancer-free women.

Altogether, the newly identified and established MD loci explained only small fractions of the total variance in volumetric MD. More information about the genetic architecture of MD can be obtained by comparing SNP-based (*h*^2^_SNP_) to family-based (*h*^2^) heritability estimates, with the missing heritability gap representing the contribution of rarer genetic variants (to be discovered by whole-genome sequencing), gene-gene or gene-environment interactions, and/or possible inflation of family-based estimates when shared environmental effects are not specified in the model, such as is the case in sibling-based designs for *h*^2^ estimation. Like other complex traits, the *h*^2^_SNP_ estimates for percent dense and absolute nondense volume were approximately half of the *h*^2^ estimates reported in our sibling study [26] (ratio *h*^2^_SNP_ to *h*^2^ = 0.46 and 0.41, respectively). Interestingly, the heritability gap was much smaller for the absolute dense volume (ratio *h*^2^_SNP_ to *h*^2^ = 0.76). Because percent dense and absolute nondense volume are highly correlated with measures of adiposity [12, 26], this finding may suggest that sibling-based *h*^2^ estimates of percent and absolute nondense volume are more likely to be confounded by shared environmental influences such as body fatness than sibling-based *h*^2^ estimates of the absolute dense volume.

To our knowledge, this is the largest genetic association study of volumetric MD to date. All mammograms were derived from the same view and analyzed using fully automated software, reducing the likelihood of random measurement error. Genotyping data were complemented with imputed variants using 1000 Genomes Project data, resulting in high genome-wide coverage. We further reproduced associations with several established MD loci. Although a fully automated method such as Volpara has clear benefits in terms of a standardized and objective MD measurement, the underlying physics model tends to underestimate MD in very dense breasts [45–47]. Together with the narrower distribution of volumetric MD compared with area-based MD, this error could have resulted in reduced statistical power to identify novel MD loci and potential lack of association in the Karma-iCOGS cohort including women with more dense breasts. Furthermore, because our study population was restricted to cancer-free women, we were unable to address the role of volumetric MD in the mediation of SNP effects on breast cancer risk.

## Conclusions

In summary, we report three novel MD loci at genome-wide significance, of which *HABP2* and *LINC01483* may represent putative new breast cancer susceptibility loci. We further demonstrate that 25% of the variance in volumetric MD is attributable to common genetic variation and that the ratio of SNP-based to narrow-sense heritability estimates varies between mammographic dense and nondense tissue components. Altogether, these findings provide more insight into the genetic basis of MD and mechanisms through which MD influences breast cancer risk.

## Additional file

Additional file 1: Supplementary Tables and Figures. (DOCX 8763 kb)

## Abbreviations

*A1*: Major allele
*A2*: Minor allele
BCAC: Breast Cancer Association Consortium
BMI: Body mass index
CTCF: CCCTC-binding factor
eQTL: Expression quantitative trait loci
ER: Estrogen receptor
GCTA: Genome-wide complex trait analysis
GWAS: Genome-wide association study
*h*^2^: Narrow-sense family-based heritability
*h*^2^_SNP_: Single-nucleotide polymorphism-based heritability
HA: Hyaluronic acid
HABP2: Hyaluronan-binding protein 2
HMEC: Human mammary epithelial cells
iCOGS: Illumina iSelect genotyping array of the Collaborative Oncological Gene-environment Study
INHBB: Inhibin beta B subunit
Karma: Karolinska Mammography Project for Risk Prediction of. Breast Cancer
KCNJ16: Potassium voltage-gated channel subfamily J, member 16
LD: Linkage disequilibrium
LINC01483: Long intergenic non-protein coding RNA 1483
MAF: Minor allele frequency
MAP2K6: Mitogen-activated protein kinase 6
MCF-7: Michigan Cancer Foundation 7
MD: Mammographic density
PCA: Principal component analysis
SNP: Single-nucleotide polymorphism
