# Supplementary material for: Common genetic variation and novel loci associated with volumetric mammographic density
Source: Breast Cancer Res. 2018 Apr 17;20:30. doi: 10.1186/s13058-018-0954-6 (PMC5904990; doi:10.1186/s13058-018-0954-6)
Supplement: Supplementary file 1 — Supplementary Tables and Figures. (DOCX 8763 kb) [file 13058_2018_954_MOESM1_ESM.docx]

**Table S1**. Sample quality control exclusions.

|  | **Number of individuals excluded** | |
| --- | --- | --- |
| **Sample QC exclusion criteria** | **Karma-OncoArray** | **Karma-iCOGS** |
| Gender discordance according to array data | 1 | 0 |
| Chromosomal anomalies (XO, XXY) | 3 | 0 |
| Discordant replicate | 0 | 1 |
| First degree relatives | 134 | 0 |
| Extreme heterozygosity | 15 | 0 |
| Low call rate | 57 | 1 |

Number of individuals excluded in the Karma study population with mammographic density data per sample QC criterion. After these exclusions, 5827 individuals with OncoArray and 4021 individuals with iCOGS array genotyping data were retained in analyses. Abbreviations: QC = quality control.

**Table S2**. Single nucleotide polymorphisms associated with volumetric mammographic density measures.

|  |  |  |  |  |  | **Karma-OncoArray**  **(N = 5827)** | | | **Karma-iCOGS**  **(N = 4021)** | | | **Pooled MD analysis** | | |
| --- | --- | --- | --- | --- | --- | --- | --- | --- | --- | --- | --- | --- | --- | --- |
| **Locus** | **BP** | **Lead SNP** | **Gene** | **A1** | **A2** | **MAF** | **Beta (SE)** | ***P* value** | **MAF** | **Beta (SE)** | ***P* value** | **Beta (SE)** | ***P* overall** | ***P* het** |
| **Percent dense volume** | | | | | | | | | | | | | | |
| 6q25.1* | 149606801 | rs4897108 | *TAB2* | G | A | 0.22 | -0.07 (0.02) | 6.4x10^-5^ | 0.18 | -0.09 (0.02) | 6.9x10^-6^ | -0.08 (0.01) | 2.5x10^-9^ | 0.44 |
| 10q21.2* | 64288130 | rs10995194 | *ZNF365* | G | C | 0.16 | -0.12 (0.02) | 2.7x10^-10^ | 0.16 | -0.10 (0.02) | 8.1x10^-6^ | -0.11 (0.01) | 1.4x10^-14^ | 0.47 |
| 10q25.3 | 115248851 | rs2089176 | *HABP2* | A | G | 0.41 | -0.05 (0.01) | 3.2x10^-4^ | 0.41 | -0.08 (0.02) | 1.4x10^-6^ | -0.07 (0.01) | 4.1x10^-9^ | 0.20 |
| **Absolute dense volume** | | | | | | | | | | | | | | |
| 2q14.2 | 121092388 | rs12468790 | *INHBB* | A | G | 0.39 | 0.08 (0.02) | 2.4x10^-5^ | 0.36 | 0.08 (0.02) | 2.3x10^-4^ | 0.08 (0.01) | 2.1x10^-8^ | 0.84 |
| 4q13.3* | 75419787 | rs10034692 | *AREG* | A | G | 0.32 | -0.10 (0.02) | 3.5x10^-7^ | 0.32 | -0.09 (0.02) | 1.0x10^-4^ | -0.09 (0.01) | 1.5x10^-10^ | 0.74 |
| 10q21.2* | 64278682 | rs10995190 | *ZNF365* | G | A | 0.15 | -0.13 (0.02) | 4.4x10^-8^ | 0.16 | -0.14 (0.03) | 1.9x10^-6^ | -0.13 (0.02) | 4.1x10^-13^ | 0.86 |
| 17q24.3 | 67836371 | rs9302903 | *LINC01483* | T | C | 0.08 | -0.12 (0.03) | 7.5x10^-5^ | 0.08 | -0.17 (0.04) | 1.3x10^-5^ | -0.14 (0.02) | 5.9x10^-9^ | 0.36 |
| 22q13.1* | 41025380 | rs6001984 | *MKL1* | A | G | 0.11 | -0.13 (0.03) | 3.2x10^-6^ | 0.12 | -0.12 (0.03) | 2.4x10^-4^ | -0.13 (0.02) | 3.0x10^-9^ | 0.85 |
| **Absolute non-dense volume** | | | | | | | | | | | | | | |
| 8p11.23 * | 36846109 | rs7816345 | - | C | T | 0.15 | -0.08 (0.02) | 1.1x10^-6^ | 0.15 | -0.07 (0.02) | 1.4x10^-3^ | -0.08 (0.01) | 6.9x10^-9^ | 0.49 |
| 22q13.1* | 41018451 | rs145351698 | *MKL1* | C | T | 0.11 | -0.08 (0.02) | 9.7x10^-5^ | 0.13 | -0.11 (0.02) | 2.9x10^-6^ | -0.09 (0.02) | 1.9x10^-9^ | 0.31 |

Betas represent change in log transformed volumetric density per minor allele increase. Abbreviations: BP = base pair position (build 37); SNP = single nucleotide polymorphism; A1 = major allele; A2 = minor allele; MAF = minor allele frequency; SE = standard error. INFO scores of index SNPs (Karma-OncoArray – Karma-iCOGS): rs4897108 (0.97-0.91); rs10995194 (1.00-1.00), rs2089176 (0.99-0.98), rs12468790 (0.96-0.92), rs10034692 (0.94-1.00), rs10995190 (1.00-1.00), rs9302903 (0.99-0.96), rs6001984 (0.99-0.98), rs7816345 (1.00-1.00), rs145351698 (0.99-0.99). * Loci identified by previous GWAS or breast cancer susceptibility variant analyses of mammographic density. Gene refers to nearest gene.

**Table S3.** Association between previously identified MD loci and volumetric mammographic density measures.

|  |  |  |  |  |  |  | **From literature** | | | **Karma-OncoArray (N = 5827)** | | | **Karma-iCOGS (N = 4021)** | | | **Pooled MD analysis** | |
| --- | --- | --- | --- | --- | --- | --- | --- | --- | --- | --- | --- | --- | --- | --- | --- | --- | --- |
| **REF** | **Locus** | **BP** | **SNP** | **Gene** | **A1** | **A2** | **MAF** | **Beta (SE)*** | ***P*** | **MAF** | **Beta (SE)** | ***P*** | **MAF** | **Beta (SE)** | ***P*** | **Beta (SE)** | ***P* overall** |
| **Percent dense volume** | | | | | | | | | | | | | | | | | |
| 1 | 1q12.21 | 149892872 | rs11205277 | *-* | A | G | 0.43 | -0.26 (0.05) | 1.3x10^-10^ | 0.43 | -0.05 (0.01) | 1.6x10^-3^ | 0.42 | -0.03 (0.02) | 0.05 | -0.04 (0.01) | 2.3x10^-4^ |
| 2 | 5q23.2 | 122454305 | rs186749 | *PRDM6* | A | G | 0.28 | 0.10 (0.02) | 2.5x10^-9^ | 0.35 | 0.03 (0.02) | 2.5x10^-2^ | 0.36 | 0.03 (0.02) | 0.15 | 0.03 (0.01) | 8.2x10^-3^ |
| 3 | 6q25.1 | 149606801 | rs9485370 | *TAB2* | G | T | 0.17 | -0.16 (0.05) | 3.9x10^-4^ | 0.18 | -0.07 (0.02) | 1.2x10^-4^ | 0.18 | -0.09 (0.02) | 2.5x10^-5^ | -0.08 (0.01) | 1.3x10^-8^ |
| 2 | 8p11.23 | 36846109 | rs7816345 | *-* | C | T | 0.18 | 0.08 (0.02) | 4.7x10^-8^ | 0.15 | 0.09 (0.02) | 3.5x10^-6^ | 0.15 | 0.02 (0.02) | 0.3 | 0.06 (0.02) | 3.1x10^-5^ |
| 4 | 10q21.2 | 64288130 | rs10995194 | *ZNF365* | G | C | 0.15 | -0.18 (0.03) | 9.6 x10^-10^ | 0.16 | -0.12 (0.02) | 2.7x10^-10^ | 0.16 | -0.10 (0.02) | 8.1x10^-6^ | -0.11 (0.01) | 1.4x10^-14^ |
| 5 | 12q24.2 | 114868138 | rs1265507 | *TBX5* | G | A | 0.48 | -0.25 (0.06) | 1.0x10^-8^ | 0.46 | -0.06 (0.01) | 5.3x10^-6^ | 0.46 | -0.01 (0.02) | 0.5 | -0.04 (0.02) | 8.9x10^-5^ |
| 2 | 22q13.1 | 38628306 | rs7289126 | *TMEM184B* | C | A | 0.40 | -0.10 (0.02) | 4.7x10^-9^ | 0.44 | 0.00 (0.01) | 0.9 | 0.45 | -0.04 (0.02) | 0.03 | -0.02 (0.01) | 0.1 |
| **Absolute dense volume** | | | | | | | | | | | | | | | | | |
| 2 | 4q13.3 | 75419787 | rs10034692 | *AREG* | A | G | 0.26 | -0.16 (0.03) | 2.1x10^-10^ | 0.32 | -0.10 (0.02) | 3.5x10^-7^ | 0.32 | -0.09 (0.02) | 1.0x10^-4^ | -0.09 (0.01) | 1.5x10^-10^ |
| 3 | 6q25.1 | 149606801 | rs9485370 | *TAB2* | G | T | 0.17 | -0.22 (0.05) | 1.0 x10^-5^ | 0.18 | -0.08 (0.02) | 5.9x10^-4^ | 0.18 | -0.08 (0.03) | 2.0x-10^-3^ | -0.08 (0.02) | 3.9x10^-6^ |
| 2 | 6q25.1 | 151946629 | rs12665607 | *ESR1* | T | A | 0.08 | 0.17 (0.04) | 1.7x10^-8^ | 0.07 | 0.06 (0.03) | 0.09 | 0.07 | 0.17 (0.04) | 7.3x10^-5^ | 0.10 (0.03) | 1.4x10^-4^ |
| 3 | 6q25.1 | 151955985 | rs60705924 | *CCDC170/ESR1* | A | G | 0.31 | 0.09 (0.04) | 0.03 | 0.31 | 0.04 (0.02) | 0.02 | 0.31 | 0.08 (0.02) | 1.9x10^-4^ | 0.06 (0.01) | 3.0x10^-5^ |
| 2 | 10q21.2 | 64278682 | rs10995190 | *ZNF365* | G | A | 0.16 | -0.24 (0.03) | 1.5x10^-16^ | 0.15 | -0.13 (0.02) | 4.4x10^-8^ | 0.16 | -0.14 (0.03) | 1.9x10^-6^ | -0.13 (0.02) | 4.1x10^-13^ |
| 2 | 11p15.5 | 1909006 | rs3817198 | *LSP1* | T | C | 0.34 | 0.14 (0.03) | 9.7x10^-11^ | 0.29 | 0.04 (0.02) | 0.03 | 0.30 | 0.01 (0.02) | 0.7 | 0.03 (0.01) | 0.06 |
| 2 | 12q23.2 | 103011894 | rs703556 | *IGF1* | A | G | 0.02 | -0.41 (0.08) | 3.7x10^-10^ | 0.03 | -0.13 (0.05) | 8.7x10^-3^ | 0.04 | -0.19 (0.06) | 9.1x10^-4^ | -0.16 (0.04) | 3.2x10^-5^ |
| 2 | 22q13.1 | 38628306 | rs7289126 | *TMEM184B* | C | A | 0.40 | -0.11 (0.02) | 2.8x10^-8^ | 0.44 | -0.03 (0.02) | 0.11 | 0.45 | -0.04 (0.02) | 0.08 | -0.03 (0.01) | 0.02 |
| 2 | 22q13.1 | 40778231 | rs17001868 | *SGSM3/MKL1* | A | C | 0.08 | -0.18 (0.03) | 2.3x10^-13^ | 0.15 | -0.08 (0.02) | 1.4x10^-3^ | 0.16 | -0.06 (0.03) | 0.05 | -0.07 (0.02) | 2.3x10^-4^ |
| **Absolute non-dense volume** | | | | | | | | | | | | | | | | | |
| 2 | 8p11.23 | 36846109 | rs7816345 | *-* | C | T | 0.18 | -0.24 (0.03) | 2.4x10^-23^ | 0.15 | -0.08 (0.02) | 1.1x10^-6^ | 0.15 | -0.07 (0.02) | 1.4x10^-3^ | -0.08 (0.01) | 6.9x10^-9^ |

Betas represent change in log transformed volumetric density per minor allele increase. Abbreviations: CHR = chromosome; BP = base pair position (build 37); SNP = single nucleotide polymorphism; A1 = major allele; A2 = minor allele; MAF = minor allele frequency; SE = standard error; REF= reference (to studies identifying genome-wide significant loci associated with mammographic density): 1 = Fernandez-Navarro et al, 2015 ^6^; 2 = Lindstrom et al, 2014 ^5^; 3 = Brand et al, 2015 ^7^; 4= Lindstrom et al, 2011 ^3^; 5 =Stevens et al, 2012 ^4^. Genes refer to nearby genes. INFO scores of SNPs (Karma-OncoArray – Karma-iCOGS): rs11205277 (1.00-1.00), rs186749 (0.99-0.97), rs9485370 (1.00-1.00), rs7816345 (1.00-1.00), rs10995194 (1.00-1.00), rs1265507 (1.00-1.00), rs7289126 (1.00-1.00), rs10034692 (0.94-1.00), rs12665607 (1.00-1.00), rs60705924 (1.00-1.00), rs10995190 (1.00-1.00), rs3817198 (1.00-1.00), rs703556 (0.99-1.00), rs17001868 (1.00-1.00)

**Table S4.** Regulatory features associated with newly identified mammographic density loci.

| **Locus** | **SNP** | **LD (r^2^)** | **MAF** | **SiPhy** | **Cons** | **Promoter histone marks** | **Enhancer histone marks** | **DNAse** | **Proteins bound** | **Motifs changed** | **Active in** |
| --- | --- | --- | --- | --- | --- | --- | --- | --- | --- | --- | --- |
| 10q25.3 | [rs11196334](http://www.broadinstitute.org/mammals/haploreg/detail_v4.1.php?query=&id=rs11196334) | 0.84 | 0.34 | x |  |  | x |  |  | x | HMEC |
| 10q25.3 | [rs1472751](http://archive.broadinstitute.org/mammals/haploreg/detail_v4.1.php?query=&id=rs1472751) | 0.88 | 0.35 |  |  |  | x | x | x |  | HMEC, MCF-7 |
| 10q25.3 | [rs17769979](http://archive.broadinstitute.org/mammals/haploreg/detail_v4.1.php?query=&id=rs17769979) | 0.84 | 0.34 | x |  | x | x |  |  | x | HMEC |
| 17q24.3 | [rs28758835](http://archive.broadinstitute.org/mammals/haploreg/detail_v4.1.php?query=&id=rs28758835) | 0.94 | 0.09 |  |  |  | x |  |  | x | HMEC |
| 17q24.3 | [rs28631707](http://archive.broadinstitute.org/mammals/haploreg/detail_v4.1.php?query=&id=rs28631707) | 0.94 | 0.09 |  |  |  | x |  |  | x | HMEC |

Candidate variants with regulatory features at newly identified mammographic density loci. Regulatory variants (lead SNP or variants in strong LD, r2 > 0.8) as identified using the HaploReg and the RegulomeDB databases. Abbreviations: SNP = single nucleotide polymorphism; LD = linkage disequilibrium; HMEC = breast mammary epithelial cells; MCF-7 = mammary tumor cells. Proteins bounds ([rs1472751](http://archive.broadinstitute.org/mammals/haploreg/detail_v4.1.php?query=&id=rs1472751)): CTCF (in HMEC and MCF-7) and GATA3 (in MCF-7).

**Figure S1.** Distributions of volumetric mammographic density measures in the Karma-OncoArray and Karma-iCOGS genotyping cohort.


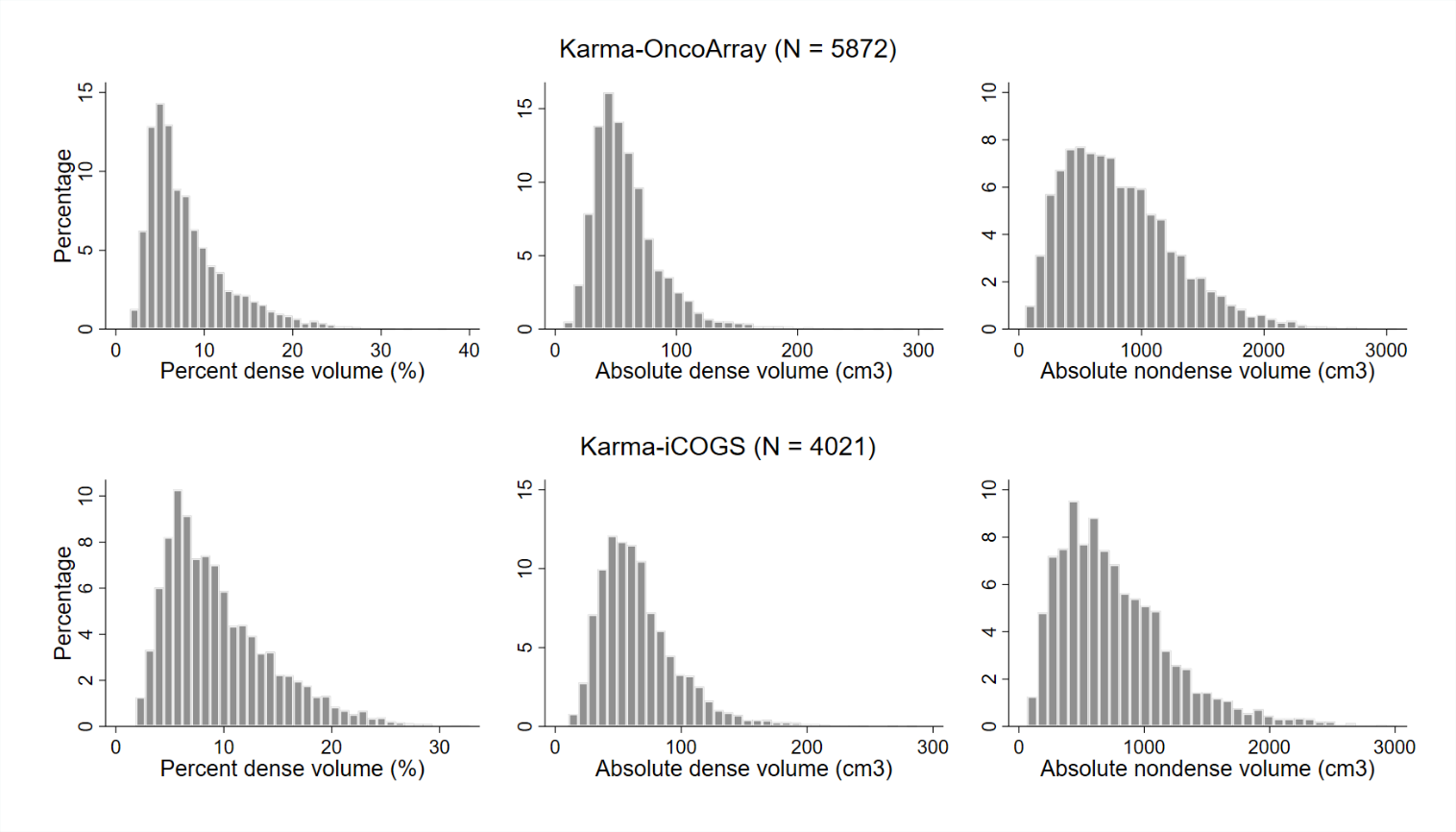


**Figure S2.** Distributions of log transformed volumetric mammographic density measures in the Karma-OncoArray and Karma-iCOGS genotyping cohort.

**
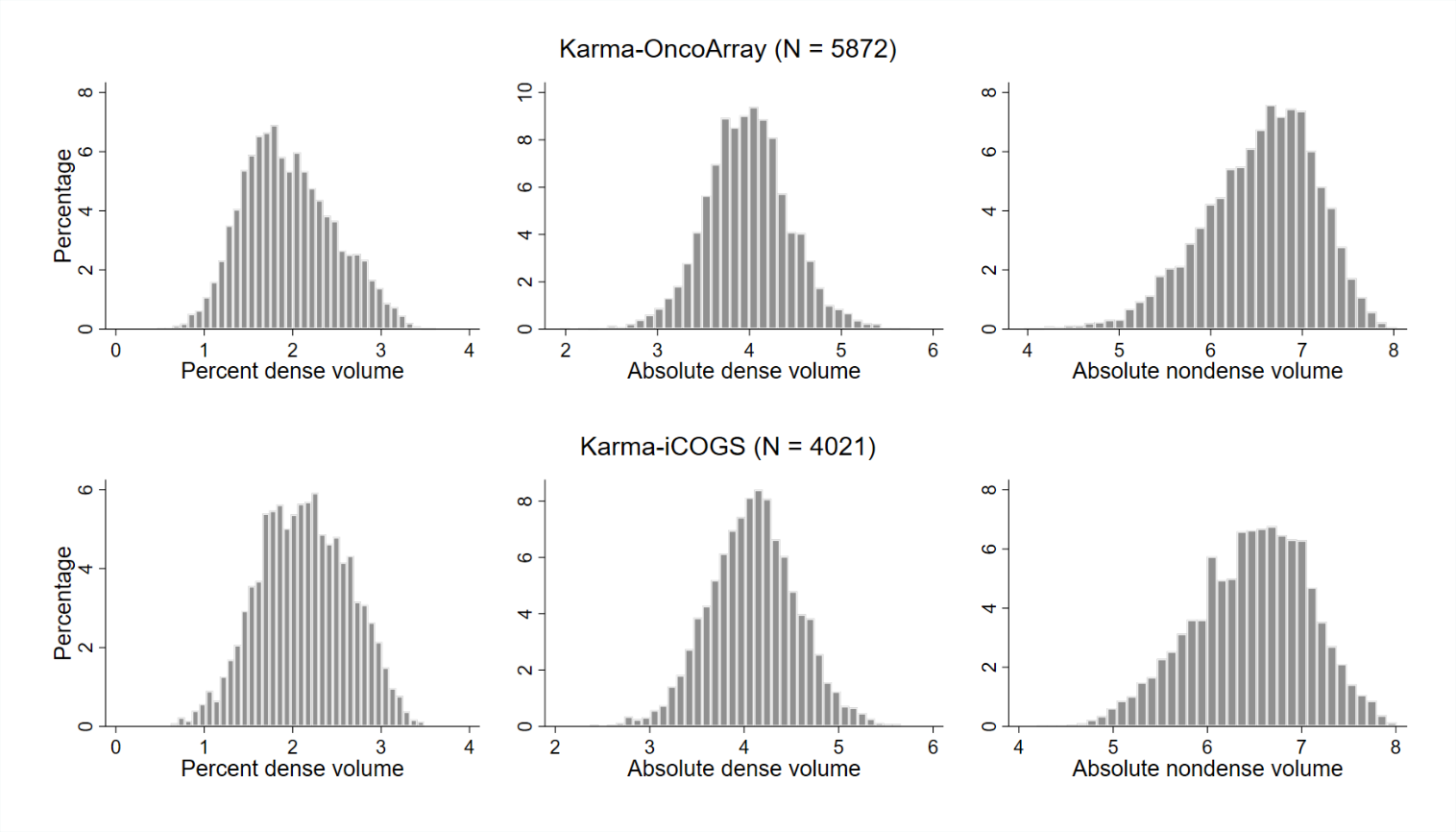
**

All volumetric mammographic measures were log-transformed prior to analyses to approximate the normal distribution.

**Figure S3.** QQ plots of association results for percent dense volume (A), absolute dense volume (B) and absolute non-dense volume (C).

**A** **B**  **C**

**
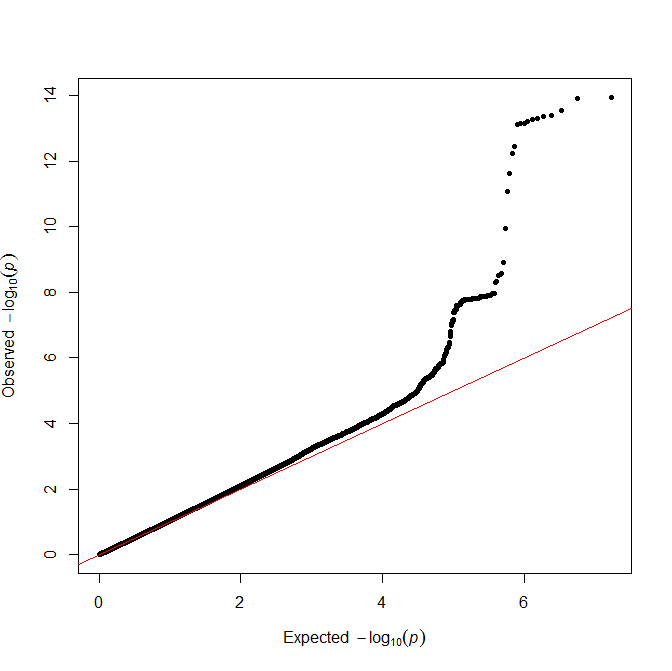

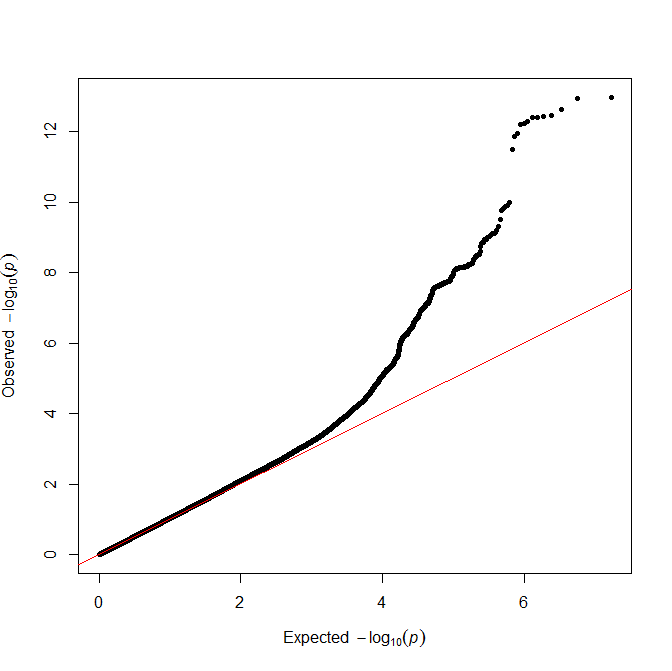

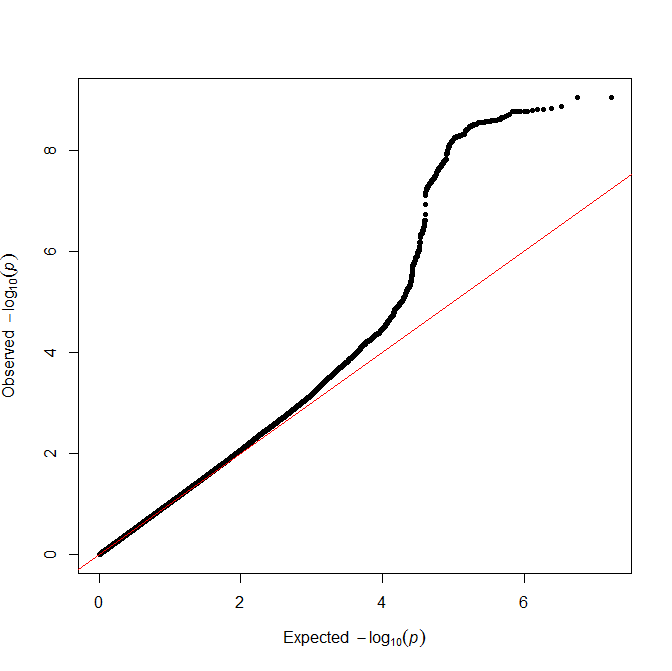
**

Quantile-quantile (QQ) plots of association results in meta-analysis of Karma-OncoArray and Karma-iCOGS data. A = percent dense volume; B = absolute dense volume; C = absolute non-dense volume. The observed P values are plotted against the expected distribution of P values under the null distribution.

**Figure S4.** Manhattan plots of associations results for percent dense volume (A), absolute dense volume (B) and absolute non-dense volume (C).

**A** **B**  **C**


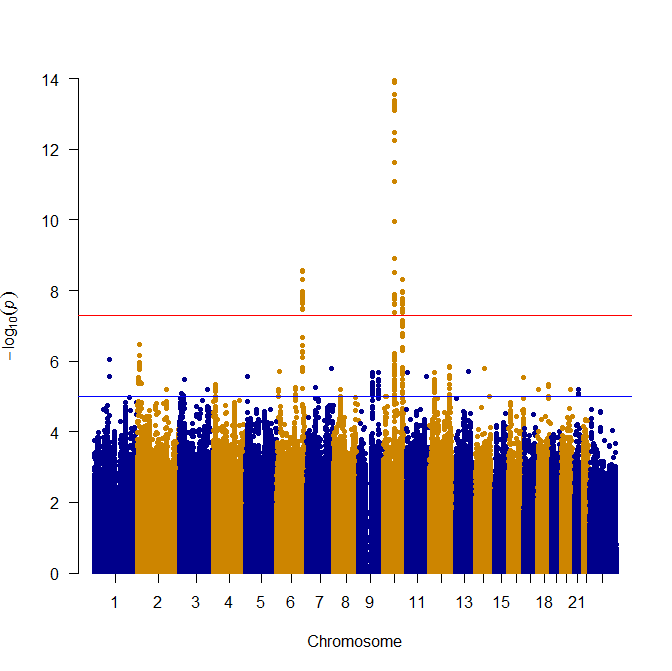

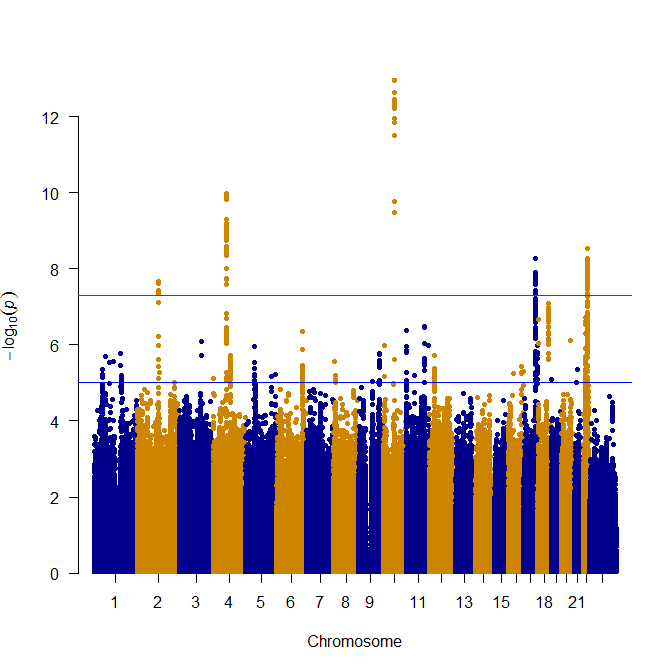

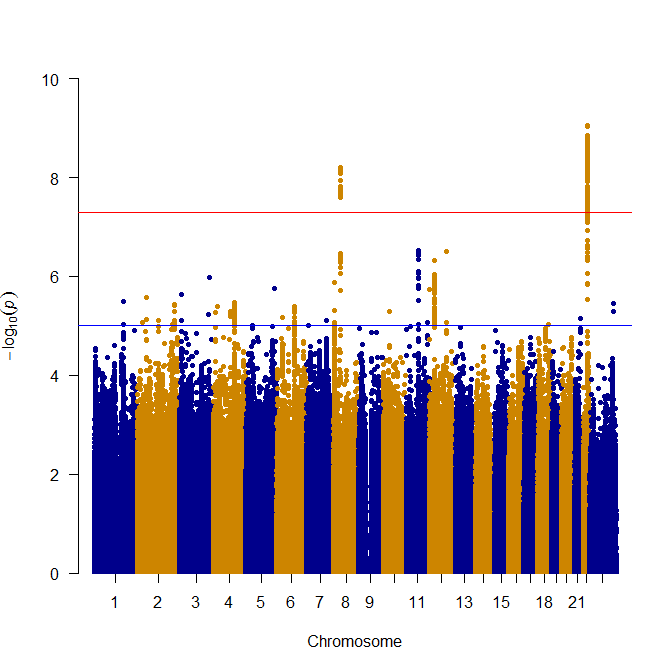


Manhattan plots of the association results in meta-analysis of Karma-OncoArray and Karma-iCOGS data, per volumetric mammographic density measure. A = percent dense volume; B = absolute dense volume; C = absolute non-dense volume. The –log10 (P) values are plotted against chromosomal base-pair position.
